# A critical factor in reactive oxygen species (ROS) studies: the need to understand the chemistry of the solvent used: the case of DMSO†

**DOI:** 10.1039/d4sc05038j

**Published:** 2024-10-08

**Authors:** Shubham Bansal, Binghe Wang

**Affiliations:** a Department of Chemistry, Center for Diagnostics and Therapeutics, Georgia State University Atlanta Georgia 30301 USA wang@gsu.edu +1-404-413-5544

## Abstract

Reactive oxygen species (ROS) play critical roles in normal physiological processes including cellular signaling and immune responses. Various pathological conditions including infections of various types, inflammation, cancer, and respiratory conditions are associated with elevated levels of ROS. Therefore, there is widespread interest in understanding ROS concentrations under various pathophysiological conditions for diagnostic and therapeutic applications including ROS-triggered drug delivery. However, in determining ROS concentration, there are major concerns of inappropriate use of various methods that lead to erroneous results; this has prompted the publication of a consensus paper in *Nature Metabolism* by a group of ROS experts stating “Unfortunately, the application and interpretation of these measurements are fraught with challenges and limitations. This can lead to misleading claims.” Along this line, we have identified an overlooked factor, which can significantly skew the results and results interpretation: the organic co-solvent. DMSO is one of the most widely used organic co-solvents to dissolve a reagent for bioassays. Herein, we describe the rapid oxidation of DMSO by hypochlorite and how this oxidation impacts results of ROS determination in buffer, cell culture media, cell culture, and cell lysates. We hope to use this one example to draw attention to the convoluted roles that DMSO and possibly other organic co-solvents can play and skew experimental results. We also hope to stimulate additional studies to bring more rigor to studying ROS concentration and biology.

## Abstract 2

Reactive oxygen species (ROS) play critical roles in normal physiological processes including cellular signaling and immune responses.

## Graphical abstract

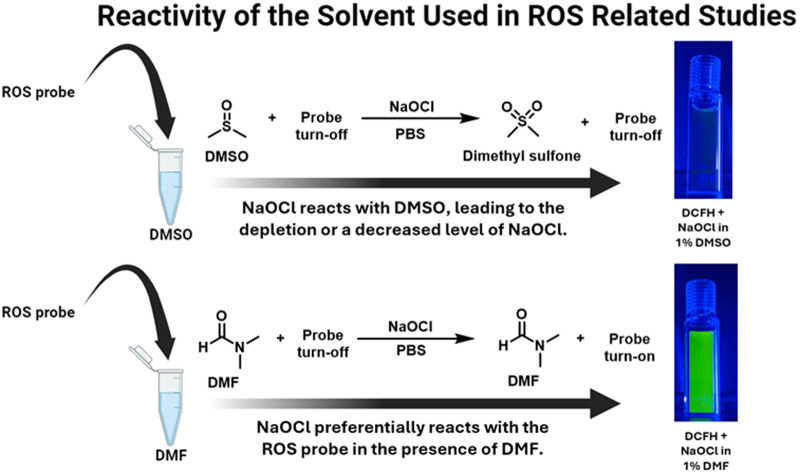

## 1. Introduction

Reactive oxygen species (ROS) play critical roles in cellular signaling, immune responses, oxidative damage, and aging, among others.^1^ Therefore, there is widespread interest in understanding ROS concentrations under various pathophysiological conditions for studying basic biological mechanistic questions and for ROS-sensitive delivery of drugs and/or imaging agents.^2–6^ Biologically relevant ROS include two general categories: (1) one-electron oxidants such as hydroxyl radical (OH˙), superoxide (O_2_^−^˙), alkoxyl radical (RO˙), nitrogen dioxide (NO_2_˙), and alkyl peroxyl (ROO˙) and (2) two-electron oxidants such as H_2_O_2_, hypochlorous acid or hypochlorite (HOCl/OCl^−^), and peroxynitrite (ONOO^−^).^7^ Among these, almost all one-electron oxidants are very short-lived including OH˙, O_2_^−^˙, RO˙, NO_2_˙, and ROO˙ because of their radical nature and high reactivity. For example, superoxide radical anion (O_2_˙^−^)^8^ spontaneously dismutates into hydrogen peroxide (H_2_O_2_) and molecular oxygen (O_2_)^9^ with a second-order rate constant 5 × 10^5^ M^−1^ s^−1^, which can be further increased to 5 × 10^9^ M^−1^ s^−1^ by superoxide dismutase (SOD).^10^ This means that any other reactions of O_2_˙^−^ must be at least faster than spontaneous dismutation in order to be kinetically relevant.^11^ Formation of peroxynitrite (ONOO^−^) from NO˙ and O_2_˙^−^ is the only reaction known to have a faster rate constant (1.9 × 10^10^ M^−1^ s^−1^).^12^ With the complexity of ROS studies, there are a few critical issues to pay attention to. First, ROS is not a single species and should be examined individually in order to understand its mechanistic significance. Second, the rapidly growing interest in ROS “has led researchers unfamiliar with the complexities of ROS and their reactions to employ commercial kits and probes to measure ROS and oxidative damage inappropriately,” as stated in a recent consensus paper in *Nature Metabolism* by a group of ROS experts.^13^ “Unfortunately, the application and interpretation of these measurements are fraught with challenges and limitations. This can lead to misleading claims entering the literature and impeding progress,” concludes this consensus paper. Third, the application of ROS-sensitive delivery of drugs^14–17^ or imaging agents^18,19^ is predicated on two key functions: (1) a sufficient magnitude of concentration difference for the interested ROS between normal tissue/cells and intended targets and (2) appropriate reaction kinetics to ensure a sufficient level of payload delivery and selectivity for the intended tissue/cells. However, the quality of the literature data and measurement methods of ROS “concentrations” vary significantly, dictating the need for analyzing whether and/or how one can benchmark or at least compare data from various publications. We have recently published a paper discussing these issues.^11^ All these indicate the need to carefully examine the chemistry issues in order to generate reliable ROS concentration data for individual species under various conditions. Such information is the foundation for understanding ROS biology at the molecular level.

In this study, we examine one overlooked issue in ROS concentration determination and ROS triggered prodrug activation, the use of an organic co-solvent in solubilizing a relevant reagent. Specifically, dimethyl sulfoxide (DMSO) is commonly used in sample preparation in ROS-related studies. For example, we surveyed 50 publications of ROS studies in the subject areas of both chemistry and biology and found DMSO usage as a co-solvent at different concentrations in about 80% of the publications. However, DMSO is rapidly oxidized by hypochlorite and other more reactive species. It should be noted that hypochlorite is the second most abundant ROS. For example, hypochlorite has been reported to be present in the concentration range of 20–85 μM in unstimulated cells such as HepG2,^20^ MCF-7,^20^ LO2,^20,21^ 298T,^21^ and HT-29.^20^ In animal model studies, alcohol-induced liver injury in mice has been reported to lead to the generation of 100 μM of hypochlorite while acetaminophen has been reported to lead to an even higher level of hypochlorite production.^11^ Therefore, ROS studies have to consider the presence of this highly abundant ROS.

We herein show in detail the rationale of the study and how the use of DMSO can significantly skew results in studying ROS generation.

## 2. Results and discussion

**Fig. 1:**
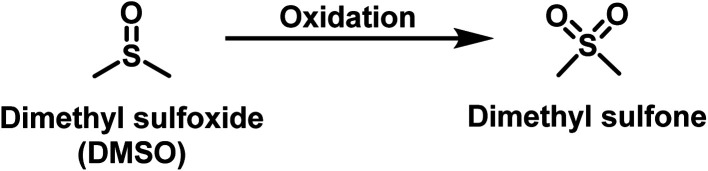
Reaction of DMSO with ROS.

For studying ROS biology, the ability to reliably determine the concentration of individual species is an important first step. Along this line, there have been many reports of very clever chemistry used for this purpose.^22–26^ Among all the ROS species, all one-electron oxidants are short-lived and do not exist at high concentration.^27^ Therefore, their production rate is more of an issue than “concentration” *per se*. However, there are two relatively stable ROS, H_2_O_2_ and hypochlorite, which do accumulate. As a result, their concentration determination is of great interest. In this regard, H_2_O_2_ is the most abundant and the least reactive among all ROS.^11^ H_2_O_2_ concentration in various pathological diseases has been reported to be as high as 610 μM.^11^ Using the oxidation of methionine as an example, the second-order rate constant is 2 × 10^−2^ M^−1^ s^−1^ for its oxidation by H_2_O_2_, which is relatively slow.^28^ The relative stable nature of H_2_O_2_ can allow reliable concentration determination chemistry under normal physiological conditions. In contrast, the second most abundant ROS, hypochlorous acid or hypochlorite (HOCl/OCl^−^), is much more reactive with a second-order rate constant of 3.7 × 10^8^ M^−1^ s^−1^ for the same oxidation of methionine.^29,30^ The reactivity of hypochlorite extends way beyond methionine. We wonder how this high reactivity of hypochlorite would impact reliability of its concentration determination using commonly used methods. We focus on the use of one co-solvent, DMSO, as an example to highlight issues that one has to be very careful in designing experiments and controls to study hypochlorite concentration. In experimental studies, we found rapid reaction between DMSO and hypochlorite under biologically relevant experimental conditions (Fig. 1). Below we describe how “low levels” of DMSO have a profound impact on experimental outcome when dealing with hypochlorite. We study this issue in buffer, cell culture media, cell culture, and cell lysates to draw attention to the convoluted roles that DMSO can play and skew experimental results.

### 2.1. Reactivity of DMSO with NaOCl

As a first step, we studied the reactivity of DMSO with NaOCl in solution using NMR with either DMSO or NaOCl in excess. In any case, DMSO concentration was kept in the mM range for a sufficient signal-to-noise ratio. To put this concentration in perspective, 1% DMSO is commonly used in cell culture experiments. This seemingly low concentration of 1% corresponds to around 140 mM of DMSO. In contrast, hypochlorite concentrations in various disease model have been observed to be up to 200 μM.^11^ This means that even a low level of 0.1%, DMSO is still in large excess compared to hypochlorite species. One would expect severe interference problem by DMSO, considering its reactivity with hypochlorite.

**Fig. 2:**
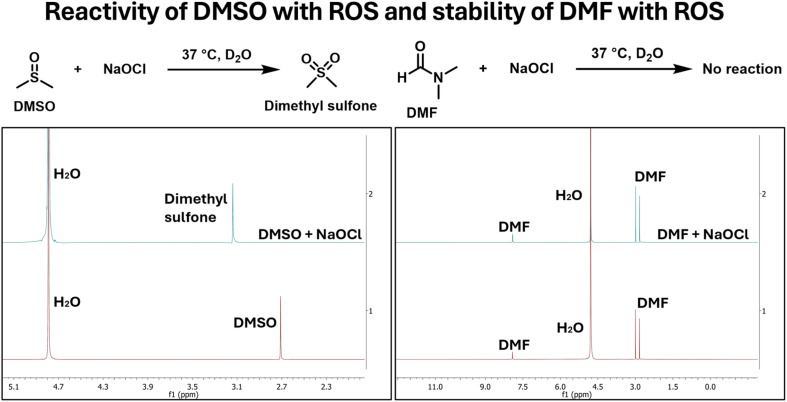
NMR spectra showing reactivity of DMSO and stability of DMF in the presence of NaOCl in the relevant time scale.

When NaOCl was in excess in the experiment, we saw complete consumption of DMSO. Briefly, when 10 mM of DMSO was incubated with 30 mM of NaOCl in D_2_O for 30 min, complete consumption of DMSO was observed as indicated by the disappearance of the signal at 2.72 ppm concomitant with the appearance of a new signal at 3.17 ppm, corresponding to dimethyl sulfone (Fig. 2a). DMF was also tested as a potential alternative using the same procedures. Similarly, 10 mM of DMF was incubated with 30 mM of NaOCl in D_2_O for 30 min. After 30 min incubation of 10 mM DMF with 30 mM of NaOCl, there was no change in the NMR signal, indicating DMF's stability within the experimental time frame.

**Fig. 3:**
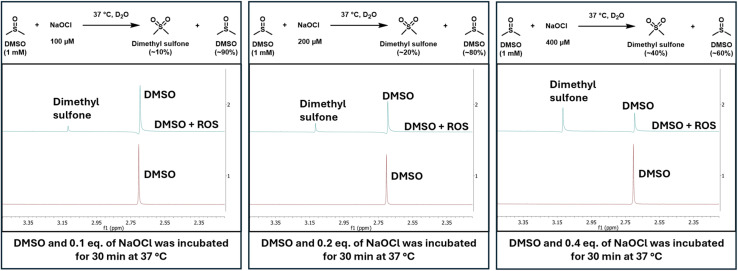
NMR spectra showing DMSO conversion to dimethyl sulfone proportional to the concentration of hypochlorite.

The second set of experiments were conducted to establish the reactivity of DMSO at physiologically relevant concentrations of hypochlorite. For this, 1 mM DMSO was incubated with NaOCl at 100 μM, 200 μM, and 400 μM (Fig. 3). We regard 400 μM of NaOCl as the upper boundary condition in cells or *in vivo*. In all the studies, DMSO concentration was kept at 1 mM, which is at the lower boundary of DMSO concentration used in normal cell culture experiments. Specifically, when 1 mM of DMSO was incubated for 30 min at 37 °C with NaOCl at various concentrations in D_2_O, DMSO was converted to dimethyl sulfone in proportion to the hypochlorite concentration (Fig. 3).

The above experiments show that ROS reaction with DMSO is a very fast process in the context of ROS concentration determination in cell culture and in biochemical assays. However, it should be noted that the fast chemical reaction between two such species has been known for a long time. It is unfortunate that this chemical reactivity issue had not been prominently highlighted in the context of biologically relevant ROS studies. The second-order rate constant for hypochlorite reaction with DMSO has been reported as 350 M^−1^ s^−1^.^31^ To put all these in biologically relevant context, even 0.01% DMSO (1.4 mM) would be in large excess of what is considered as a high hypochlorite concentration (140 μM). If one assumes pseudo first-order kinetics, the *t*_1/2_ for hypochlorite consumption would be 1.4 s leading to almost complete consumption within 10 s. If the results in solution hold true in cell culture, one would expect significant interference problems in the presence of DMSO. In discussing the biologically relevance of DMSO interference issue, it is important to keep in mind that there are other even stronger oxidizing species produced in cellular biochemical processes. For example, the second-order rate constant is 1.0–9.86 × 10^9^ M^−1^ s^−1^ for the reaction between DMSO and ·OH, which is higher than that of HOCl with a benchmark substrate methionine (3.7 × 10^8^ M^−1^ s^−1^).^30,32,33^ As such, the *t*_1/2_ is less than a second for the reaction between hydroxy radical and DMSO even at a low concentration of 10 nM each. Therefore, one can expect interference of DMSO in the concentration determination of not only hypochlorite, but also other more reactive species such as hydroxy radical.

### 2.2. Fluorescent probe's reactivity with ROS in the presence and absence of DMSO

**Fig. 4:**
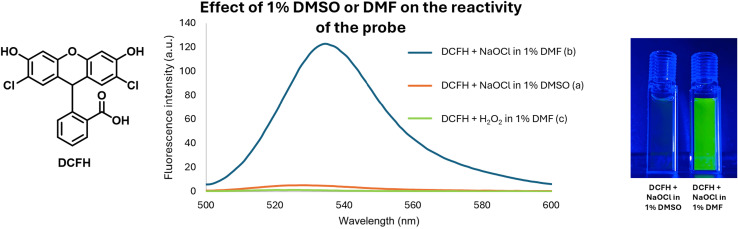
Effect of 1% DMSO or DMF on the ability for H_2_O_2_ and hypochlorite to turn on DCFH fluorescence. (a) Reaction of DCFH (10 μM) with NaOCl (100 μM) in PBS with 1% DMSO at pH 7.4 & 37 °C. (b) Reaction of DCFH (10 μM) with NaOCl (100 μM) in PBS with 1% DMF at pH 7.4 & 37 °C. (c) Reaction of DCFH (10 μM) with H_2_O_2_ (100 μM) in PBS with 1% DMF at pH 7.4 & 37 °C.

**Fig. 5:**
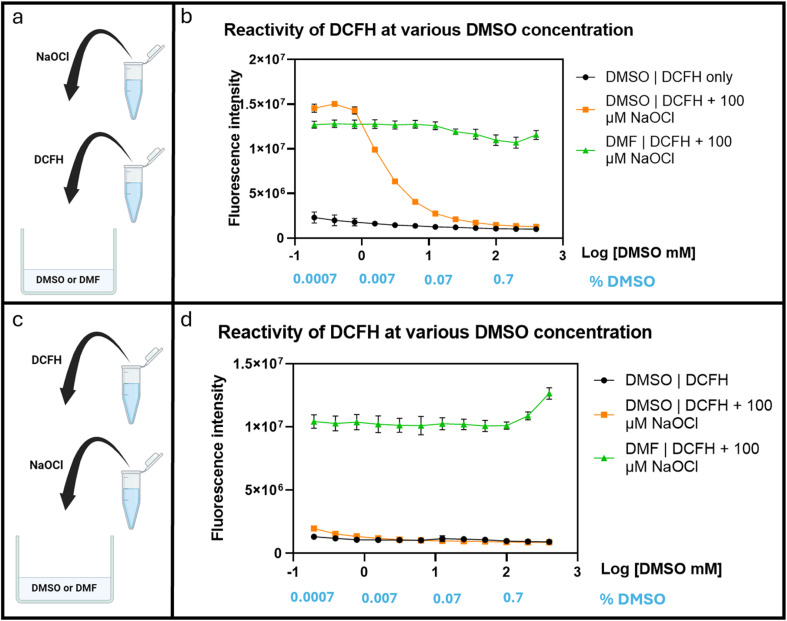
Fluorescence changes of DCFH (10 μM) in response to hypochlorite (100 μM) in the presence of DMSO or DMF at varying concentrations (0.2–400 mM). (a and b) When DCFH was added before NaOCl. (c and d) When NaOCl was added before DCFH.

After studying the reactivity of DMSO with hypochlorite in solution, we next examined how the presence of DMSO would interfere with the ability for a commonly used fluorophore, 2,7-dichlorodihydrofluorescein (DCFH) to determine hypochlorite concentration.^13^ Specifically, stock solutions of DCFH in DMSO and DMF were prepared. Then, we studied the reaction of 10 μM of DCFH and 100 μM of NaOCl at 37 °C in 1% DMF or DMSO in PBS (pH 7.4). Fig. 5 shows the stunning contrast between the fluorescence intensity of these two sets of experiments taken immediately after mixing. Specifically, the DCFH fluorescence was turned on immediately upon mixing with hypochlorite in the presence of DMF. However, the presence of DMSO only led to minimal or no fluorescence intensity changes. It is well known that DCFH does not react with H_2_O_2_.^13^ Therefore, we also used H_2_O_2_ in a comparative study as a negative control following the same procedures. As expected, DCFH only reacted quickly with NaOCl but not with H_2_O_2_ (Fig. 4).

### 2.3. DCFH reactivity with NaOCl at different DMSO concentrations

After confirming the interference of DMSO in hypochlorite reaction of DCFH, we were interested in understanding the concentration-dependence of DMSO's effect on ROS determination. Briefly, DCFH (10 μM) was dissolved in PBS at pH 7.4 with DMF or DMSO at varying concentrations (0.2–400 mM). Then NaOCl was added to achieve a final concentration of 100 μM. A 96-well plate was used for this experiment. The fluorescence intensity was recorded after incubation at 37 °C for 30 min using a plate reader (*λ*_ex_ 495 nm and *λ*_em_ 530 nm). Fig. 5 shows significant attenuation of fluorescence signal strength by DMSO starting at about 1.5 mM (Fig. 5b). This was not observed with DMF, indicating a lack of interference by DMF. Though, the interference by DMSO was clear, the high concentration (1.5 mM) needed to affect the test with only 100 μM NaOCl was surprising because one would expect very pronounced effect of DMSO in a 1 : 1 ratio with NaOCl. We reasoned that the seemingly attenuated effect of DMSO was probably due to faster reaction of HOCl with DCFH than with DMSO and thus strong competition for the available hypochlorite species. We examined this aspect by reversing the order of mixing all the components as described below.

In an experiment to study the effect of DMSO using a slightly different procedure, we first added hypochlorite to the PBS containing varying DMSO concentrations (0.2–400 mM) before DCFH addition. Under such conditions, fluorescence attenuation started at 0.2 mM of DMSO (Fig. 5d). Such results indicate the strong interference of DMSO under normally used cell culture conditions even if only 0.01% (1.4 mM) of DMSO is used. Such results also are consistent with the scenario of the reaction of hypochlorite with DCFH being faster than with DMSO. It should be noted that there have been reports of DMSO having antioxidant effect by up-regulation of the *SOD*, *GPx*, and *CAT* genes.^34^ Therefore, in cell culture experiments, there may be an extra layer of complexity because of DMSO's biological effects. For this, we were interested in studying the effect of DMSO on fluorescent studies in cell culture using DCFH-DA. We started the experiments by first conducting similar experiments in a cell-culture medium, DMEM as described in the next section.

### 2.4. DCHF-DA stability in cell culture media

**Fig. 6:**
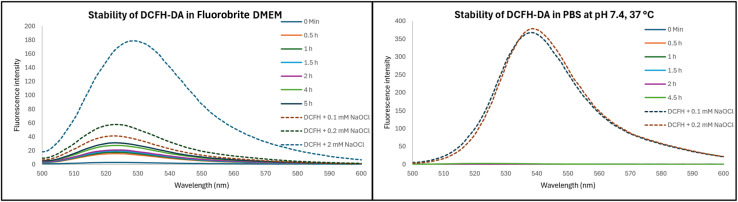
Stability of DCFH-DA in DMEM and PBS at pH 7.4 and at 37 °C.

In conducting cell culture-related work, we used DCFH diacetate (DCFH-DA), which is the diester version of DCFH meant to allow for cellular permeability. Subsequent hydrolysis of the ester groups would lead to DCFH, which is not readily cell membrane permeable and thus can be trapped intracellularly. We first checked the stability of DCFH-DA in DMEM and PBS. Briefly, a stock solution of 2 mM DCFH-DA was prepared in DMF and NaOCl solutions of 100 mM and 10 mM were prepared in H_2_O. Then 20 μM of DCFH-DA was prepared in Fluorobrite DMEM, which was then incubated at 37 °C. At designated time points, the fluorescence spectrum was recorded. When 20 μM of DCFH-DA was incubated in Fluorobrite DMEM at 37 °C, significant fluorescence increase was observed after 30 min (Fig. 6). The intensity came to about 75% of that observed after addition of hypochlorite at 0.1 mM. This is quite a significant background fluorescence. In contrast, no stability issue was observed in PBS. Such results indicate the need to consider the complexity of cell culturing medium in experimental design and in conducting control experiments. Further, it is interesting to note that the fluorescence intensity differences when different concentrations (0.1 and 0.2 mM) of hypochlorite was used. Theoretically, both should be able to consume all the DCFH in solution (20 μM). Such difference in fluorescence intensity indicates consumption of hypochlorite by component(s) of the cell culture medium, which contains organic molecules such as amino acids (*e.g.* methionine).^30^ It should be noted that the second-order rate constant of NaOCl reaction is 3.7 × 10^8^ M^−1^ s^−1^ with methionine and 10^4^ M^−1^ s^−1^ with amines.^30^ Additional evidence for this is provided by the much stronger fluorescence intensity of the same experiments when conducted in PBS, which does not have organic molecules that might consume hypochlorite (Fig. 6). In DMEM, 20 μM DCFH and 2 mM NaOCl showed intensity of about 50% of 20 μM DCFH and 0.2 mM NaOCl in PBS. Such results also indicate that DCFH-DA can be prepared and stored in PBS for a few hours but not in DMEM.

### 2.5. Effects of DMSO on ROS measurements in RAW264.7 cells

**Fig. 7:**
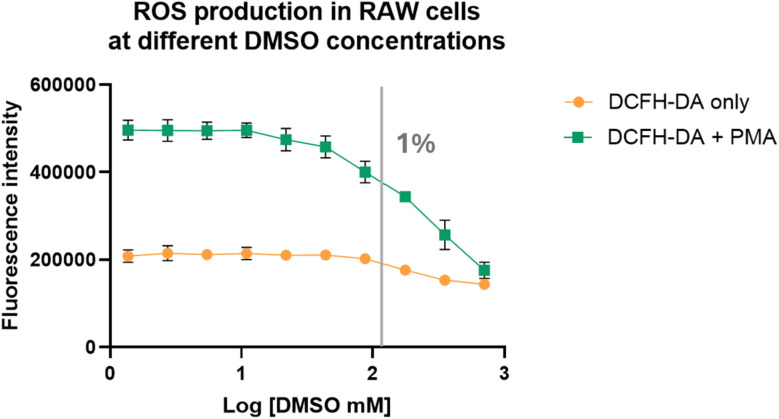
DMSO's effect on ROS production in RAW264.7 cells.

With the added layer of complexity of hypochlorite's reaction with component(s) of cell culture medium DMEM, we became interested in studying the effect of DMSO on hypochlorite concentration studies in cell culture. In doing so, we chose RAW264.7 cells (purchased from ATCC, Virginia, USA) because this cell line is often used in studies of inflammatory responses.^35^ Therefore, we used DCFH-DA to measure ROS levels in RAW264.7 cells at various DMSO concentrations with and without stimulation by PMA, a molecule known to induce inflammatory responses and thus ROS production.^36^ Briefly, RAW264.7 cells were incubated with 10 μM of DCFH-DA in the presence of DMSO at different concentrations (0.002–5% or 0.3–700 mM) with and without 1 μM of PMA. The plates were incubated for 2 h before fluorescence measurements using a plate reader. Indeed, DMSO affected the fluorescence intensity of the cell culture in a dose-dependent manner, but only marginally at high concentrations (1% or 140 mM) (Fig. 7). It is possible that the less than pronounced effect of DMSO is partially due to the basal level fluorescence as a result of incubation in cell culture medium as shown in Fig. 6. However, even with that consideration, the effect of DMSO on the fluorescence response seemed to be small compared to what was observed in water and cell culture medium. Specifically, studies in PBS buffer with 0.1% DMSO consumed almost all of the ROS. However, in cell culture studies, the effect was visible at a concentration higher than 1% DMSO. For the experiments after PMA stimulation, the effect of DMSO seemed to be much more pronounced (Fig. 7). However, even this is only at a high concentration (1% or 140 mM DMSO). The seemingly muted effects of DMSO below 1% in cell culture experiments were puzzling. It turned out that DMSO has been reported to increase ROS production by damaging mitochondrial integrity and membrane potential when studied using astrocytes.^37^ Specifically, swelling of mitochondria was observed when astrocytes were treated with 1% DMSO for 24 h. About 35% of mitochondria exhibited loss of cristae or formed monolayer vacuoles after exposure to 5% DMSO. When DMSO reacts with ROS, it forms stoichiometric amount of dimethyl sulfone, which has been reported to reduce the mitochondrial membrane potential of both cancerous and noncancerous cells^38,39^ and has been shown to attenuate toxin-induced reductions in SOD, CAT, and glutathione peroxidase (GPx) as well as to reduce myeloperoxidase (MPO) activity.^40–43^ All these present a very convoluted picture for the effect of DMSO on ROS concentration. In order to deconvolute the effect of DMSO on the cell's ability to produce ROS from that of ROS detection using DCFH, we conducted additional experiments using cell lysates, which presumably do not have the same functional cellular machineries as intact cells.

#### 2.5.1. Effect of DMSO on ROS detection using DCFH in the cell lysates

**Fig. 8:**
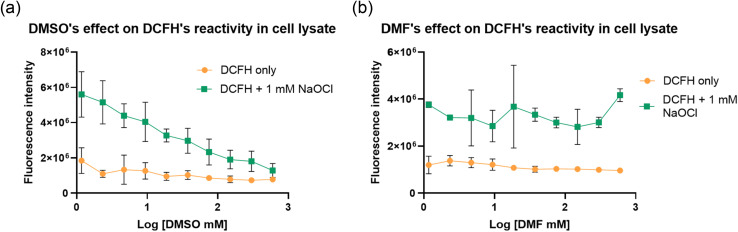
(a) Reaction of DMSO with ROS in the cell lysate. (b) DMF's effect on probe detection of ROS. DMF as one of the potential alternatives to DMSO as the DMF does not reacts with ROS in the relevant time scale.

Briefly the cells were lysed using RIPA buffer and used to study the effect of DMSO on DCFH's ability to detect ROS. The experiments were conducted in a 96-well plate, which was prepared by having a different concentration of DMSO in each well. Then cell lysate and DCFH (10 μM final concentration) were added. At last, NaOCl was added to each well to achieve a final concentration of 1 mM. The plate was then incubated for 30 min at 37 °C before fluorescence intensity measurement using a plate reader. As can be seen from Fig. 8, the results were similar to that of the experiments in buffer (Fig. 5), showing pronounced fluorescent intensity decreases at low concentrations of DMSO. Such results indicate the significant interference of DCFH's ability by DMSO in the presence of various cellular components. However, the biological effect of DMSO (and possibly dimethyl sulfone) on cellular redox activity is too convoluted to allow for deciphering on the chemical effect of DMSO.

As a potential alternative to the DMSO, DMF was tested similarly using cell lysates using the same experimental procedures. Indeed, the results were similar to that from experiments in buffer (Fig. 5), showing little concentration-dependent fluorescent intensity change (Fig. 8). Such results are consistent with the relative inert nature of DMF in the presence of hypochlorite on the time scale of ROS related studies. These results demonstrate the feasibility for DMF to be used as an alternative for solution-phase and extracellular studies. To use DMF as an alternative in cell culture or *in vivo* studies, one needs to pay attention to cellular toxicity as well. Along this line, it has been reported that the LD_50_ of DMF in rats and mice is in the range of 2.2–7.5 g kg^−1^, demonstrating safety under normal experimental conditions.^44–46^ It should be noted that LD_50_ of DMSO is 16 g kg^−1^ in mice.^47^ Though the LD_50_ value is higher for DMSO, both are considered very safe in lab experiments. In cell culture studies, the IC_50_ values for DMF and DMSO was tested in RAW-264.7, MCF-7 and HUVEC cells.^48^ Specifically, the IC_50_ values of DMSO and DMF were found to be similar in RAW-264.7, MCF-7 and HUVEC cells, in the range of 1.8–1.9% (v/v) and 1.1–1.2% (v/v), respectively.^48^ Cell viability was found to be 80% at 0.5% (v/v) of DMSO and 70% at 0.5% (v/v) of DMF.^48^ All these results support DMF being a viable alternative to DMSO in ROS-related studies in solution, cell culture, and animal models.

## 3. Conclusion

A 2022 consensus paper by a group of ROS experts has brought to the forefront of the issue of robustness of the ROS concentration data in the literature. A key issue among them is the appropriateness of the experimental conditions used and associated interpretation of results. Herein, we have described the serious problems one has to pay attention to when DMSO is used as a co-solvent because of its extensive chemical reactivity with ROS. Studies were conducted using the second most abundant ROS, hypochlorite in buffer, cell culture media, cell culture and cell lysates. We conclude that DMSO in commonly used concentrations cause severe interference problems. We would like to use this one example to highlight the need to carefully examine the chemistry issues associated with ROS concentration determination. Further, there are other alternatives for studies in buffer, cell culture media, or cell lysates: DMF. However, we did not examine the biological effect of DMF in live cells, which can be an entirely different set of issues.

## Data availability

The data associated with this article is available in the article and ESI.†

## Author contributions

Conceptualization: S. B., B. W.; data curation: S. B.; investigation: S. B.; supervision: B. W.; writing – original draft: S. B., B. W.; writing – review & editing: S. B., B. W.

## Conflicts of interest

There are no conflicts to declare.

## Supplementary Material

SC-OLF-D4SC05038J-s001
