# Supplementary material for: A critical factor in reactive oxygen species (ROS) studies: the need to understand the chemistry of the solvent used: the case of DMSO
Source: Chem Sci. 2024 Oct 8. Online ahead of print. doi: 10.1039/d4sc05038j (PMC11469295; doi:10.1039/d4sc05038j)
Supplement: SC-OLF-D4SC05038J-s001 [file SC-OLF-D4SC05038J-s001.pdf]

Supplementary Information (SI) for Chemical Science.
This journal is © The Royal Society of Chemistry 2024

Supplementary Information

A Critical Factor in Reactive Oxygen Species (ROS) Studies: The Need to Understand the Chemistry of the Solvent Used: The Case of DMSO

Shubham Bansal and Binghe Wang*

Department of Chemistry and Center for Diagnostics and Therapeutics, Georgia State University, Atlanta, Georgia 30301 USA

*Address correspondence to
Dr. Binghe Wang
Regents Professor and Dr. Frank Hannah Chair
Georgia Research Alliance Eminent Scholar
Department of Chemistry
Georgia State University
Atlanta, Georgia 30301
USA
wang@gsu.edu
Phone: 404-413-5544

1. Experimental section

1.1. Materials and Methods

All reagents and solvents were of reagent grade from Sigma-Aldrich (Massachusetts, USA) or Oakwood Products, Inc. (South Carolina, USA). 2',7'-Dichlorodihydrofluorescein diacetate, DCHFH-DA (CAS 4091-99-0 and Catalog No. HA-7023) was purchased from Combi-Blocks (California, USA). 2,7-Dichlorodihydrofluorescein, DCFH (Synonym: DCFH2, CAS 106070-31-9, and Catalog No. HY-153006) was purchased from MedChemExpress (New Jersey, USA). PMA (CAS 16561-29-8 and Catalog No. HY-18739) was purchased from MedChemExpress (New Jersey, USA). RAW264.7 cells were purchased from ATCC (Virginia, USA). ^{1}H NMR (400 MHz) spectra were recorded on a Bruker Avance 400 MHz NMR spectrometer in deuterated solvent from Oakwood Products, Inc. (South Carolina, USA). Chemical shifts were reported as δ values (ppm). TMS (δ = 0.00 ppm) or residual peaks of the deuterated solvent were used as the internal reference. Samples of milligram-quantities were weighed on C-33 microbalance (CAHN instruments Inc., California, USA). Plate reader: excitation wavelength 495 nm, emission wavelength 535 nm.

1.2. NMR studies

Firstly, 100-mM stock solutions of DMSO or DMF were prepared in D_2O. For 100-mM DMSO stock solution, 7.1 µL of DMSO was added to 992.9 µL of D_2O. For the 100-mM DMF stock solution, 7.75 µL of DMF was added to 992.25 µL of D_2O. Commercial NaOCl was used directly without purification (1.811 M). 100 mM of NaOCl was prepared by adding 55 µL of NaOCl (from 1.811 M) to 945 µL of H_2O. After that 100 µL of NaOCl (from 100 mM) to 900 µL of H_2O which gave a 10 mM working solution.

For reaction mixtures having 3 eq. of NaOCl and 1 eq. of DMSO or DMF: 50 µL (from 100-mM stock solution) of DMSO/DMF solution was added to 441.8 µL of D_2O followed by the addition the solution 8.2 µL of NaOCl (from 1.811 M). The resulting mixture was incubated at 37 °C for 30 min before acquisition of NMR spectra. Solutions of 10-mM of DMSO or DMF were prepared by adding 50 µL (from 100 mM stock) to 450 µL of D_2O.

For reaction mixtures having 1 mM of DMSO or DMF and limiting amount of NaOCl:

a) 1 mM DMSO and 100 μM of NaOCl: 5 μL of 100-mM stock solution of DMSO was added to 490 μL of D_2O followed by the addition of 5 μL of a 10-mM NaOCl solution. The resulting mixture was incubated at 37 °C for 30 minutes before NMR acquisition.
b) 1 mM DMSO and 200 μM of NaOCl: 5 μL of 100-mM stock solution of DMSO was added to 485 μL of D_2O followed by the addition of 10 μL of a 10-mM NaOCl solution. The resulting mixture was incubated at 37 °C for 30 minutes before NMR acquisition.
c) 1 mM DMSO and 400 μM of NaOCl: 5 μL of 100-mM stock solution of DMSO was added to 475 μL of D_2O followed by the addition of 20 μL of a 10-mM NaOCl was added. The resulting mixture was incubated at 37 °C for 30 min minutes before NMR acquisition.

1.3. Effect of DMSO on the reaction of fluorescent Probe with ROS

1.3.1. Effect of 1% DMSO or DMF on the reactivity of the probe

Stock solutions were prepared as

a) 10 mM DCFH in DMSO, 1.544 mg of DCFH was dissolved in 382.9 μL of DMSO. Then 1 mM working solution was prepared by adding 100 μL of DCFH (10 mM in DMSO) to 900 μL of DMSO.
b) 10 mM DCFH in DMF, 1.649 mg of DCFH was dissolved in 408.9 μL of DMF. Then 1 mM working solution was prepared by adding 100 μL of DCFH (10 mM in DMF) to 900 μL of DMF.
c) 10 mM NaOCl stock solution: First, a 100 mM of NaOCl was prepared by adding 55 μL of NaOCl (from 1.811 M) to 945 μL of H_2O. After that 100 μL of NaOCl (from 100 mM) to 900 μL of H_2O which gave a 10 mM working solution.
d) 10 mM H_2O_2 stock solution: First, a 100 mM of H_2O_2 was prepared by adding 9.09 μL of H_2O_2 (from 11 M) to 991 μL of H_2O. After that 100 μL of H_2O_2 (from 100 mM) to 900 μL of H_2O which gave a 10 mM working solution.

Sample Preparation

a) 10 μM DCFH and 100 μM NaOCl in 1% DMF in PBS: Firstly, 15 μL of DCFH (from 1 mM stock solution in DMF) was added to 1.470 mL of PBS at pH 7.4, followed by the addition of 15 μL of NaOCl (10 mM). Then the fluorescence spectrum was recorded.

b) 10 μM DCFH and 100 μM NaOCl in 1% DMSO in PBS: Firstly, 15 μL of DCFH (from 1 mM stock solution in DMSO) was added to 1.470 mL of PBS at pH 7.4 followed by the addition of 15 μL of NaOCl (10 mM). Then the fluorescence spectrum was recorded.

c) 10 μM DCFH and 100 μM H_2O_2 in 1% DMF in PBS: Firstly, 15 μL of DCFH (from 1 mM stock solution in DMF) was added to 1.470 mL of PBS at pH 7.4 followed by the addition of 15 μL of H_2O_2 (10 mM). Then the fluorescence spectrum was recorded.

1.3.2. Concentration-dependent effects of DMSO on the reactivity of the fluorescent probe

Stock solutions were prepared as

a) 1.2 M solution of DMSO was prepared by adding 172 μL of pure DMSO to 1828 μL of PBS at pH 7.4.

b) 1.2 M solution of DMF was prepared by adding 186 μL of pure DMF to 1814 μL of PBS at pH 7.4.

c) 30 μM DCFH in PBS was prepared by adding 30 μL of DCFH (10 mM in DMF) to 9.970 mL of PBS at pH 7.4.

d) 30 μM DCFH in PBS was prepared by adding 30 μL of DCFH (10 mM in DMSO) to 9.970 mL of PBS at pH 7.4.

e) 300 μM NaOCl in PBS was prepared by adding 30 μL of NaOCl (100 mM in H_2O) to 9.970 mL of PBS at pH 7.4.

Sample preparation: To conduct this set of experiments, 96-well plate (8 × 12) were used. Firstly, 200 μL of 1.2 M DMF or DMSO was added to the wells in Column 1 and the rest of wells were filled with 100 μL of PBS. Then serial dilution was conducted by starting with transferring 100 μL from each well of Column 1 to the next column. Subsequent dilutions were conducted the same way. For the last column, 100 μL of the final solution was removed and discarded. Then 100 μL of DCFH and 100 μL of NaOCl were added to each well, resulting in a total volume of 300 μL in each well and DMF/DMSO concentrations of 400 mM, 200 mM, 100 mM, 50 mM, 25 mM, 12.5 mM, 6.25 mM, 3.13 mM, 1.56 mM, 0.78 mM, 0.39 mM, and 0.20 mM. Subsequently, the plate was incubated at 37 °C for 30 min before fluorescence measurements using a plate reader. All experiments were conducted in triplicates.

1.4. Stability of DCFH-DA in cell culture media and PBS

Stock solutions were prepared as

a) 2 mM DCFH-DA in DMF: Firstly 5 mM of DCFH-DA in DMF was prepared by dissolving 1.160 mg in 476.1 µL of DMF. Then 200 µL of DCFH-DA (from 5 mM) was added to 800 µL of DMF to give a 2 mM DCFH-DA solution.

b) 100 mM and 10 mM of NaOCl solutions: First, a 100 mM of NaOCl was prepared by adding 55 µL of NaOCl (from 1.811 M) to 945 µL of H_2O. Then adding 100 µL of the NaOCl stock solution (100 mM) to 900 µL of H_2O which gave a 10 mM solution.

To monitor the stability of DCFH-DA

a) 20 µM of DCFH-DA in Fluorobrite DMEM: 15 µL of DCFH-DA (from 2 mM) was added to 1485 µL of Fluorobrite DMEM. The resulting mixture was incubated at 37 °C. At designated time points, the fluorescence spectra were recorded.

   As reference, two new solutions containing NaOCl were prepared:

   a. 20 µM of DCFH and 100 µM of NaOCl in Fluorobrite DMEM: 15 µL of DCFH (from 2 mM) was added to 1470 µL of Fluorobrite DMEM followed by the addition of 15 µL of NaOCl (from 10 mM). The fluorescence spectrum of the resulting solution was recorded.

   b. 20 µM of DCFH and 200 µM of NaOCl in Fluorobrite DMEM: 15 µL of DCFH (from 2 mM) was added to 1455 µL of Fluorobrite DMEM followed by the addition of 30 µL of NaOCl (from 10 mM). The fluorescence spectrum of the resulting solution was recorded.

   c. 20 µM of DCFH and 2 mM of NaOCl in Fluorobrite DMEM: 15 µL of DCFH (from 2 mM) was added to 1455 µL of Fluorobrite DMEM followed by the addition of 30 µL of NaOCl (from 100 mM). The fluorescence spectrum of the resulting solution was recorded.

b) 20 µM of DCFH-DA in PBS: 15 µL of DCFH-DA (from 2 mM) was added to 1485 µL of PBS. The resulting mixture was incubated at 37 °C. At designated time points, the fluorescence spectrum was recorded.

   As reference, two solutions containing NaOCl was prepared:

a. 20 μM of DCFH and 100 μM of NaOCl in PBS: 15 μL of DCFH (from 2 mM) was added to 1470 μL of PBS followed by the addition of 15 μL of NaOCl (from 10 mM). The fluorescence spectrum of the resulting solution was recorded.
b. 20 μM of DCFH and 200 μM of NaOCl in PBS: 15 μL of DCFH (from 2 mM) was added to 1455 μL of PBS followed by the addition of 30 μL of NaOCl (from 10 mM). The fluorescence spectrum of the resulting solution was recorded.

1.5. Cell studies

1.5.1. ROS production in RAW264.7 at different concentrations of DMSO

Firstly, stock solutions were prepared in DMSO.

a) 5 mM of DCFH-DA in DMSO was prepared by dissolving 0.931 mg in 382.1 μL of DMSO.
b) 1 mM of PMA in DMSO was prepared by dissolving 0.458 mg in 742.5 μL of DMSO.

c) 20 μM of DCFH-DA was prepared by adding 20 μL of 5 mM DCFH-DA to 4.980 mL of DMEM.
d) 20 μM DCFH-DA with 2 μM PMA was prepared by adding 20 μL of 5 mM DCFH-DA and 10 μL of 1 mM PMA in 4.970 mL of DMEM.

To conduct this set of experiments, two 96-well plate (8 × 12) were used. The first plate was used to prepare DCFH-DA with and without PMA having various concentrations of DMSO. Firstly, 180 μL of media and 20 μL of DMF or DMSO was added to the wells in Column 1 and the rest of wells were filled with 100 μL of PBS. Then serial dilution was conducted by starting with transferring 100 μL from each well of Column 1 to the next column. Subsequent dilutions were conducted the same way. For the last column, 100 μL of the final solution was removed and discarded. Then 100 μL of DCFH-DA (20 μM) with and without PMA (2 μM) was added to the designated wells. Subsequently, a second plate 96-well plate having RAW264.7 cells was prepared to have over 80% confluency. From the cell containing plate media was removed and 120 μL from each well of first plate was transferred to the plate containing RAW264.7 cells. The RAW264.7 cells were finally incubated having 10 μM DCFH-DA with and without 1 μM PMA and the DMSO concentrations of 700 mM, 350 mM, 175 mM, 87.5 mM, 43.75 mM, 21.8 mM, 10.9 mM, 5.4 mM,

2.7 mM, 1.3 mM, 0.6 mM, and 0.30 mM. Subsequently, the plate was incubated 2 h before fluorescence measurements using a plate reader. All experiments were conducted in triplicates.

1.5.2. DMSO's reaction with ROS in cell lysate system

Stock solutions were prepared as

a) 1.2 M solution of DMSO was prepared by adding 172 µL of pure DMSO to 1828 µL of PBS at pH 7.4.
b) 1.2 M solution of DMF was prepared by adding 186 µL of pure DMSO to 1814 µL of PBS at pH 7.4.
c) 80 µM DCFH in PBS was prepared by adding 160 µL of DCFH (10 mM in DMF) to 19.840 mL of PBS at pH 7.4.
d) 80 µM DCFH in PBS was prepared by adding 160 µL of DCFH (10 mM in DMSO) to 19.840 mL of PBS at pH 7.4.
e) 8 mM NaOCl in PBS was prepared by adding 88 µL of NaOCl (1.811 mM) to 19.912 mL of PBS at pH 7.4.

Cell lysate preparation: RAW264.7 cells were used to prepare cell lysate. 2 mL of 2.3×10^7 cells/mL RAW264.7 cells were centrifuged to prepare cell pallet. After centrifuge, the supernatant was discarded, and the cells were washed by PBS (2×2 mL). After removing the PBS, two mL of RIPA buffer was added to the cell pallet to prepare cell lysate. Then, 0.5 mL of this cell lysate was added to 11.5 mL of PBS which was then used in the experiment.

To conduct this set of experiments, 96-well plate (8 × 12) were used. Firstly, 200 µL of 1.2 M DMF or DMSO was added to the wells in Column 1 and the rest of wells were filled with 100 µL of PBS. Then serial dilution was conducted by starting with transferring 100 µL from each well of Column 1 to the next column. Subsequent dilutions were conducted the same way. For the last column, 100 µL of the final solution was removed and discarded. Then 25 µL of (80 µM) DCFH. Then, 50 µL of cell lysate was added to each well. Finally, 25 µL of 8 mM NaOCl was added to each well. Each well had 10 µM of DCFH and 1 mM of NaOCl. Each well resulting in a total volume of 200 µL and DMF/DMSO concentrations of 600 mM, 300 mM, 150 mM, 75 mM, 37.5 mM, 18.75 mM, 9.38 mM, 4.69 mM, 2.34 mM, 1.17 mM, 0.59 mM, and 0.29 mM. Subsequently,

the plate was incubated at 37 °C for 30 min before fluorescence measurements using a plate reader. All experiments were conducted in triplicates.
